# The Impact of Etiology on Time to Vocal Fold Motion Recovery in Unilateral Vocal Fold Paralysis

**DOI:** 10.1002/lary.70586

**Published:** 2026-04-28

**Authors:** Rishi Suresh, Ted Mau

**Affiliations:** ^1^ Clinical Center for Voice Care, Department of Otolaryngology‐Head and Neck Surgery University of Texas Southwestern Medical Center Dallas Texas USA; ^2^ Division of Laryngology, Department of Otolaryngology‐Head and Neck Surgery Stanford University School of Medicine Palo Alto California USA

**Keywords:** idiopathic vocal fold paralysis, intubation‐related vocal fold paralysis, recurrent laryngeal nerve injury, vocal fold motion recovery, vocal fold paralysis

## Abstract

**Objectives:**

Spontaneous recovery of vocal fold (VF) motion in unilateral vocal fold paralysis (UVFP) has been shown to follow a bimodal time distribution for peripheral, nonidiopathic etiologies, but recovery time dependencies on UVFP etiology are incompletely characterized. This study aimed to (1) determine recovery time differences between surgically induced, idiopathic, and intubation‐related UVFP, and (2) investigate the impact of distance of injury from larynx on recovery time in surgically induced UVFP.

**Methods:**

Retrospective study of 1425 UVFP patients over a 16‐year period identified 736 patients with recovery of VF motion. The time to the first laryngoscopic sign of VF motion recovery in surgically induced, idiopathic, or intubation‐related UVFP was fit to a bimodal or unimodal mathematical model. Time course of VF motion recovery was also compared using Kaplan–Meier estimates.

**Results:**

Surgically induced UVFP demonstrated a bimodal time‐to‐recovery distribution, consistent with a previously described two‐phase model of recovery. Both idiopathic and intubation‐related UVFP followed unimodal recovery patterns. Among surgically induced cases, Kaplan–Meier recovery curves for thyroid/parathyroid and cardiothoracic surgeries were nearly identical despite differing injury locations along the recurrent laryngeal nerve.

**Conclusion:**

Differences in the time course of VF motion recovery in UVFP reflect the underlying etiologies. Among surgically induced UVFP, the recovery time did not correlate with the distance between the injury site and the larynx, challenging a long‐held assumption. The findings support a model in which the severity of neural injury, not the location of injury, is the principal determinant of time to recovery.

**Level of Evidence:**

3.

## Graphical abstract

The time course of vocal fold motion recovery in UVFP reflects the underlying etiologies. This study showed that there was no difference in the time to recovery between UVFP from cardiothoracic versus thyroid/parathyroid surgeries, challenging the long‐held assumption that recovery time is proportional to the distance between injury site and larynx. The findings support a model in which the severity of neural injury, not the location of injury, is the principal determinant of time to recovery.

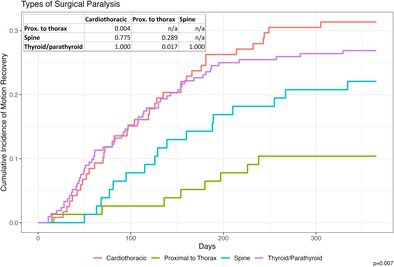

## 1 Introduction

Spontaneous recovery from unilateral vocal fold paralysis (UVFP) has been shown to follow a nonlinear time course. For peripheral, nonidiopathic causes, both recovery of voice and recovery of vocal fold (VF) motion follow a bimodal time distribution [1, 2, 3]. An early group reflects recovery from milder recurrent laryngeal nerve (RLN) injuries (e.g., Sunderland first‐ and second‐degree [4]). A late group likely corresponds to recovery from more severe injuries, with a “latent” phase presumably reflecting a rate‐limiting step to reestablish neural connectivity at the site of injury [1]. In contrast, voice recovery in idiopathic UVFP does not demonstrate a bimodal distribution and is better modeled as a unimodal phenomenon [2]. It is assumed that recovery of VF motion in idiopathic UVFP would also be unimodal, but how its time course compares with other UVFP etiologies (e.g., surgically induced) has not been characterized. Another group of interest are those patients with intubation‐related UVFP. A large‐scale study showed this group had a median recovery time of 4.3 months [5]. It is not known how the recovery time profile compares with those of other etiologies. A better delineation of how UVFP etiology impacts recovery time would provide more precise information for patient counseling and expectation setting.

In addition to defining the recovery differences between the broad categories of surgically induced, idiopathic, and intubation‐related UVFP, this work also aimed to investigate differences between subtypes of surgeries that lead to UVFP. It has been long assumed that recovery time after RLN injury is proportional to the distance between the injury site and the larynx along the nerve. Our previous work strongly suggested otherwise [1], a conclusion that was further supported by other clinical studies [6, 7] and experiments in a canine model of RLN injury [8]. However, more direct clinical evidence is needed to refute this time‐honored assumption.

This study set out to address two questions. First, how does the VF motion recovery time course differ among patients with surgically induced, intubation‐related, and idiopathic UVFP? While our prior study established that patients with idiopathic UVFP recover *voice* with a different time‐to‐recovery distribution from those with iatrogenic UVFP, this study used a larger dataset to quantify the differences in time to *VF motion* recovery among these three subgroups. We hypothesized that idiopathic and intubation‐related UVFP follow a unimodal recovery model, distinct from the bimodal model that described recovery from surgically induced UVFP. Second, how does the time course of VF motion recovery vary according to the site of injury along the RLN? We hypothesized that the time course would not follow the traditional linear prediction based on injury location.

## 2 Methods

### 2.1 Subjects and Data Collection

This study was approved by the UT Southwestern Institutional Review Board (STU 042014‐011, STU‐2024‐0030). Informed consent was waived given its retrospective nature. This work utilized the same dataset collected in Suresh et al. [3] but entailed new data analysis. In brief, a chart review was conducted of all patients diagnosed with UVFP between 2008 and 2024 at a tertiary care academic laryngology practice. Laryngoscopy videos from all visits were reviewed to determine a consensus degree of VF motion on a four‐level grading scale as detailed in Suresh et al. [3]: No motion (Grade 0), trace or flicker of purposeful motion (Grade 1), more than trace and less than half of normal motion (Grade 2), near normal or normal range of motion (Grade 3). Patients with VF paresis were excluded. To be included in VF motion recovery analysis, patients must have presented within 1 year of the onset of UVFP, and the time between a laryngoscopy showing no VF motion and a subsequent laryngoscopy showing some return of motion must be 180 days or less. The latter criterion puts an upper bound on the lag between the exam finding of VF motion and the actual onset of return of VF motion.

The date of surgery was used as the date of onset for surgically induced and intubation‐related UVFP. For idiopathic UVFP, only patients who reported onset of vocal symptoms to within a week of precision were included, consistent with our previous work [1, 2].

### 2.2 Data Modeling

The time to VF motion recovery was modeled as detailed previously [1, 3]. Briefly, an exponentially modified Gaussian distribution (ex‐Gaussian) [9] was utilized because its parameters can be interpreted in physiologically meaningful terms to reflect the processes that may underlie recovery from UVFP. For surgically induced UVFP, the ex‐Gaussian was used to model the late group, and a simple Gaussian was added to model the early group. This bimodal distribution was fit to the time to earliest noted VF motion in MATLAB utilizing a maximum likelihood estimation fitting algorithm. For idiopathic and intubation‐related UVFP, the same bimodal model was fit to the data first. If the fitting did not converge or if the fitted curve did not have an obvious bimodal distribution, then the unimodal, ex‐Gaussian fit was attempted. See S1 for details of mathematical modeling and data fitting.

### 2.3 Statistical Testing

Statistical analyses were performed in R (R Core Team, Version 4.4.1) using the Survival package (Version 3.8‐3). Kaplan–Meier recovery curves were created to estimate the time to event for the relevant subgroups. A global log‐rank test was performed to determine if an overall difference existed. If positive, post hoc pairwise comparisons using log‐rank test were then performed with Bonferroni correction.

## 3 Results

**FIGURE 1:**
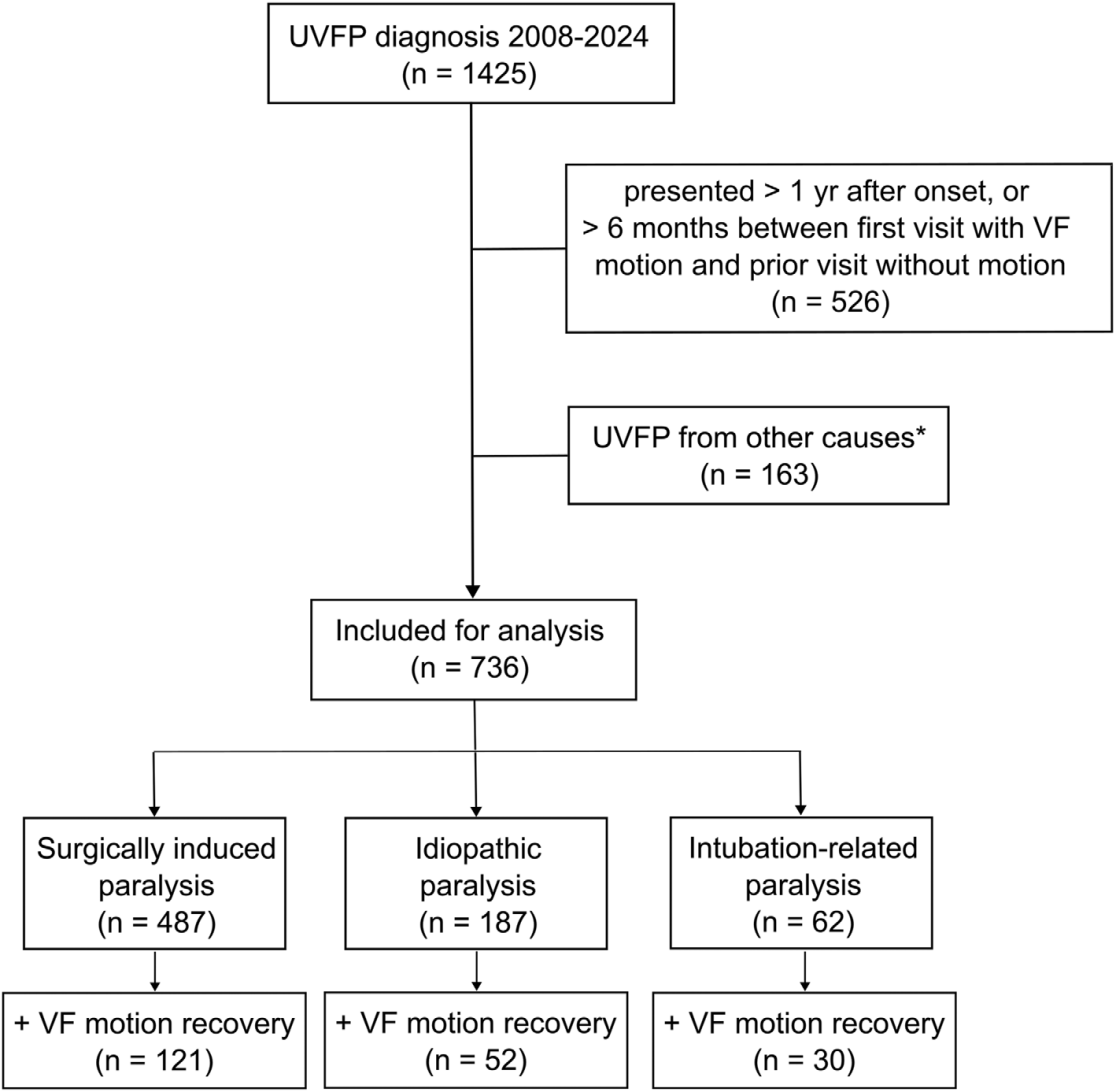
Study flow diagram. Other causes of UVFP excluded from analysis included tumor compression/invasion of RLN/vagus, neck abscess/infection, other neck mass, neurologic, Ortner syndrome, sarcoidosis, and trauma.

The same starting dataset of 1425 patients over a 16‐year period reported in Suresh et al. [3] was used for new data analysis. The study flow diagram is shown in Figure 1. Demographic details of the final included cohort are shown in Table 1. Seven hundred and thirty‐six patients had some degree of return of VF motion. Four hundred and eighty‐seven of these had surgically induced UVFP, and their specific surgical etiologies and categorizations are shown in Table 2.

**TABLE 1:** Demographics of patients included for VF motion recovery analysis.

	Surgically induced (*n* = 487)	Idiopathic (*n* = 187)	Intubation‐related (*n* = 62)	All etiologies (*n* = 736)
Age, median (IQR), years	60 (48–70)	64 (51–72)	63 (55–73)	61 (49–71)
Sex, male/female (%)	41.3%/58.7%	46.5%/53.5%	32.2%/67.8%	41.8%/58.2%
Left‐sided UVFP (%)	58.9%	65.8%	66.1%	61.3%

**TABLE 2:** Types of surgeries that led to UVFP.

	*n*	Region^a^	RLN vs. vagus^b^
Thyroid/parathyroid	212	Thyroid/parathyroid	RLN
Cardiothoracic	105	Cardiothoracic	RLN
Spine	77	Spine	RLN
Carotid	32	Proximal to thorax	Vagus
Skull base/craniotomy	19	Proximal to thorax	Vagus
Neck dissection	16	Proximal to thorax	Vagus
Esophageal	13	Cardiothoracic	RLN
Vagus nerve surgery^c^	10	Proximal to thorax	Vagus
Diverticulum	2	Not included	RLN
Cricoid resection	1	Not included	RLN
Total	487		

^a^ As included in Figure 4.

^b^ Most likely nerve involved.

^c^ Vagal nerve stimulators and vagal paraganglioma resection without nerve sacrifice.

**FIGURE 2:**
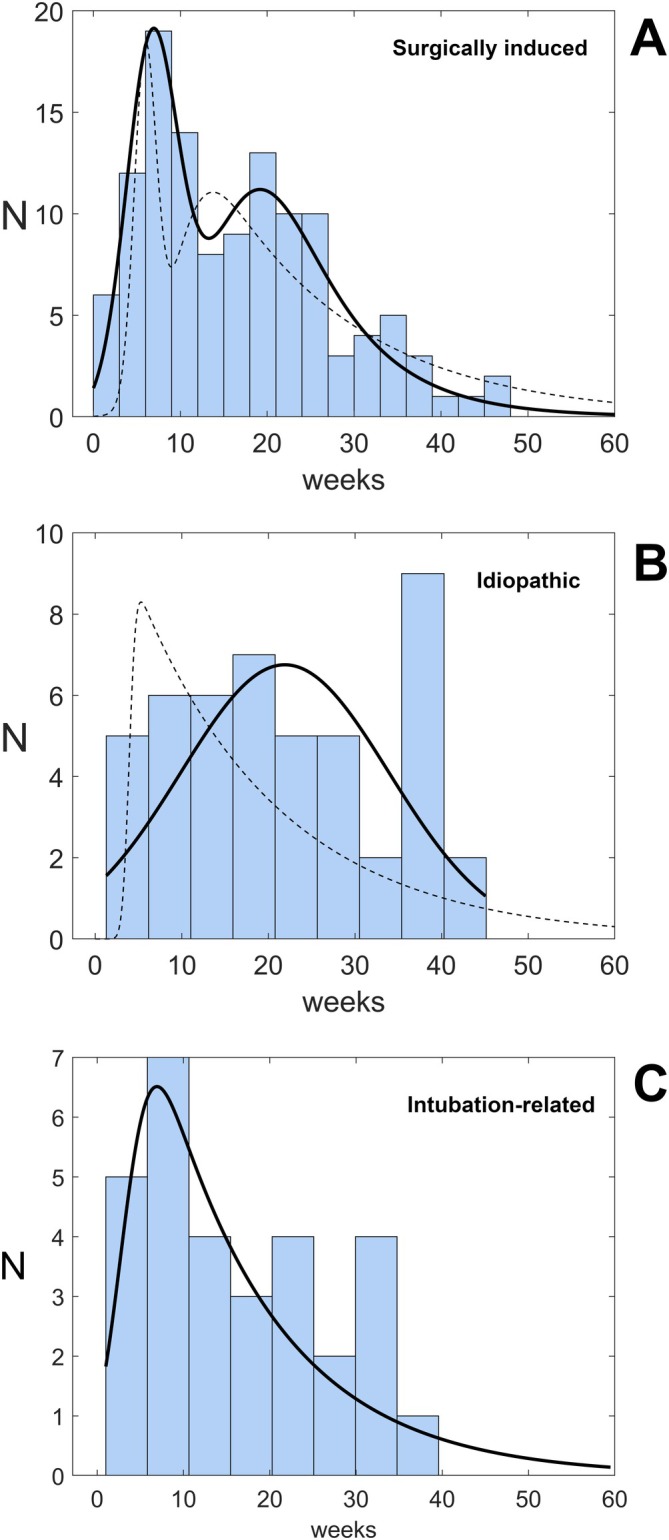
Histograms of time to initial VF motion recovery for (A) surgically induced, (B) idiopathic, and (C) intubation‐related UVFP. Thick black curve represents best fit of the model to the time to VF motion recovery data. In (A), thin dashed curve shows voice recovery from our prior work, modeled only with data from surgically induced UVFP [2]. In (B), thin dashed curve reproduces the voice recovery curve in idiopathic UVFP from Ref. [2]. [Color figure can be viewed in the online issue, which is available at www.laryngoscope.com]

The time‐to‐VF motion recovery data were fit to either a bimodal model or a unimodal model. Recovery times for surgically induced UVFP fit well to a bimodal model (Figure 2A), consistent with our prior work. Recovery times for idiopathic UVFP did not fit a bimodal model and were fit to a unimodal model (Figure 2B). Similarly, recovery times for intubation‐related UVFP clearly fit a unimodal model (Figure 2C). Note that in each case, the model was fit to the underlying data that made up the histogram, not the histograms themselves. For surgically induced UVFP and idiopathic UVFP, voice recovery curves from our prior work are also shown for comparison. There was insufficient data to model voice recovery for intubation‐related UVFP.

Since a continuous mathematical model now described the time to recovery, a cumulative probability of recovery could be calculated at any time point. Table 3 lists such probabilities at specific time points. For example, for those with idiopathic UVFP who will eventually recover VF motion, about 81% would have recovered by 6 months. Similarly, for those with intubation‐related UVFP who will eventually recover VF motion, about 51% would have done so by 3 months.

**TABLE 3:** Cumulative probabilities of recovering VF motion.

	3 months	4 months	6 months	9 months	12 months
Surgically induced	42.0%	55.3%	81.2%	96.3%	99.3%
Idiopathic	20.1%	32.7%	61.9%	91.9%	99.4%
Intubation‐related	50.9%	64.4%	81.5%	93.0%	97.4%

*Note:* Probabilities are based on a denominator consisting of those who eventually recover VF motion.

**FIGURE 3:**
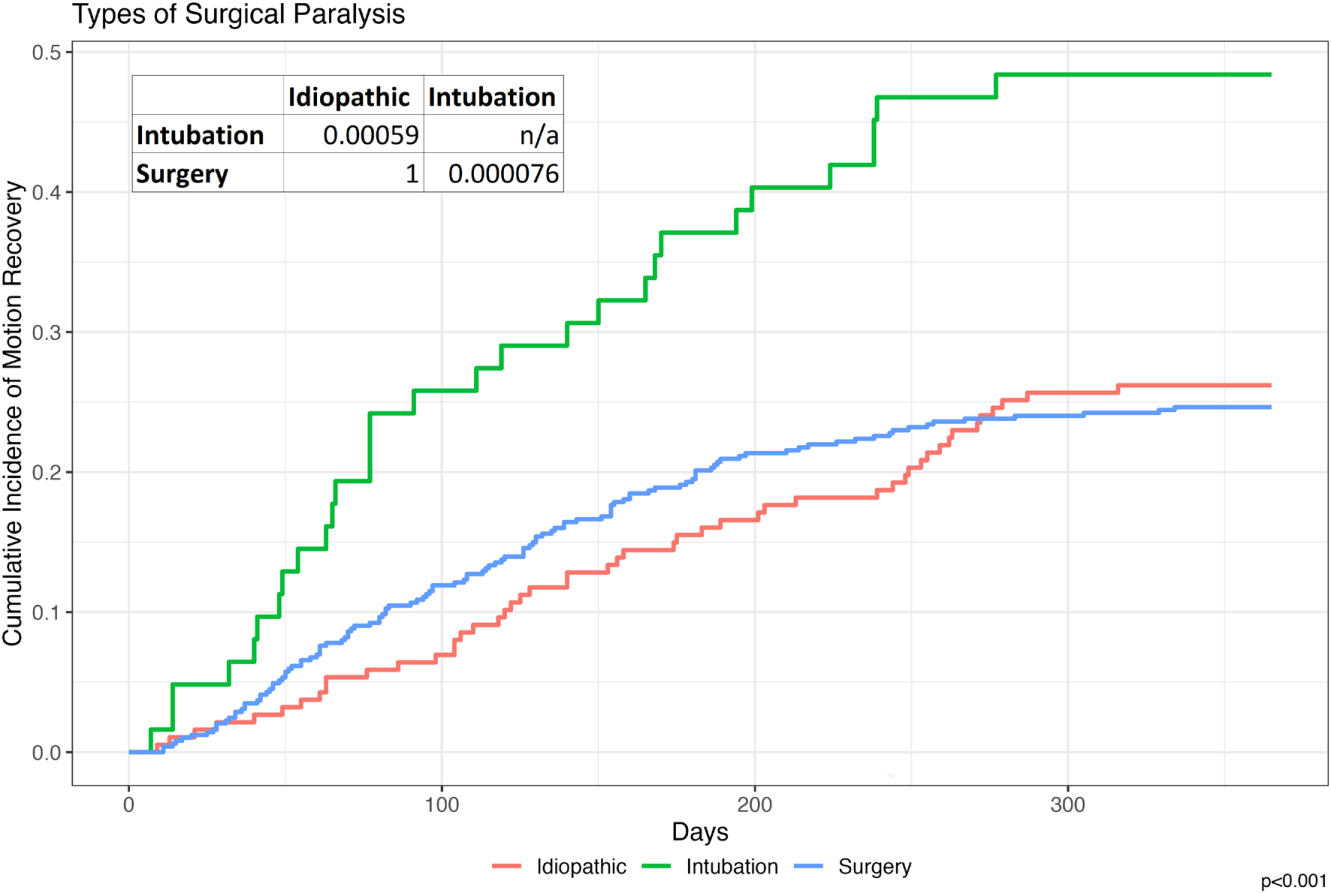
Kaplan–Meier recovery curves for VF motion recovery of any grade (Grade 1, 2, or 3) for surgically induced (blue), idiopathic (red), and intubation‐related (green) UVFP. Table shows the Bonferroni‐adjusted *p* values. Bonferroni‐adjusted *α* = 0.0167. [Color figure can be viewed in the online issue, which is available at www.laryngoscope.com]

The estimated cumulative incidence of any VF motion recovery is shown in Figure 3 as Kaplan–Meier recovery curves. Note that each curve is a continuous step function that represents every data point, while the curves in Figure 2 represent mathematical models fit to the data, so the two may not be perfectly congruent. Also note that the absolute rate of recovery on the *y*‐axis is necessarily an underestimate because not all patients had follow‐up extending to a year. The comparison between curves is more meaningful than the absolute rates of recovery. The proportion of patients who eventually recover VF motion with either surgically induced or idiopathic UVFP was approximately half of those with intubation‐related UVFP. The Kaplan–Meier curves estimate that intubation‐related UVFP has the fastest median time to recovery at 91 days (95% CI, 63–194), with surgically induced paralysis at a median of 107 days (95% CI, 81–132) and idiopathic with a median of 153 days (95% CI, 110–244). The difference between intubation‐related and surgically induced paralysis was statistically significant, as was the difference between intubation‐related and idiopathic (box inset, Figure 3).

**FIGURE 4:**
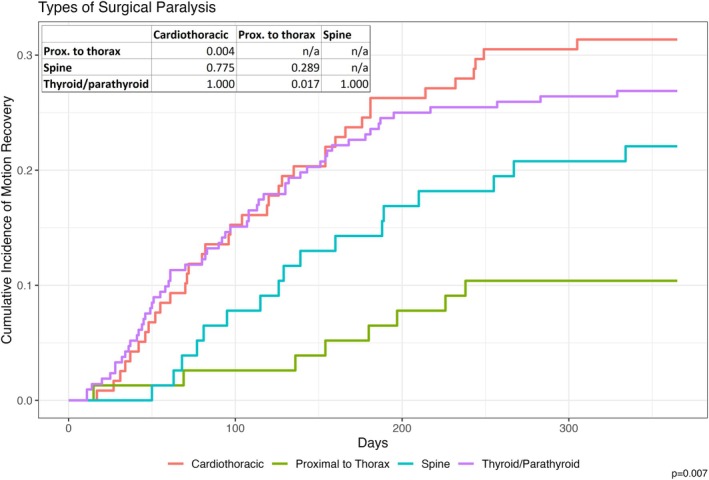
Kaplan–Meier recovery curves for VF motion recovery of any grade (Grade 1, 2, or 3) as a function of type/location of surgery: Cardiothoracic (red), proximal to thorax (green), spine (blue), and thyroid/parathyroid (purple). Table shows the Bonferroni‐adjusted *p* values. Bonferroni‐adjusted *α* = 0.0083. [Color figure can be viewed in the online issue, which is available at www.laryngoscope.com]

Figure 4 estimates the cumulative incidence of any VF motion recovery from surgically induced paralysis based on the type/location of injury. Again, note that the relative rates of recovery are more meaningful than the absolute rates, which are necessarily underestimates. Patients with UVFP from cardiothoracic and thyroid/parathyroid surgeries have very similar rates of VF motion recovery, with essentially overlapping time courses within the first 5 months. Spine surgery‐related UVFP has an intermediate rate of recovery, and surgery proximal to the thorax along the course of the RLN or vagus nerve has the lowest rate of VF motion recovery. The latter is not surprising since the proximal‐to‐thorax group mostly consisted of injuries to the vagus nerve (Table 2). The median time to VF motion recovery was 90 days (95% CI, 60–114) for thyroid/parathyroid surgery, 104 days (95% CI, 72–154) for cardiothoracic surgery, 129 days (95% CI, 95–210) for spine surgery, and 167 days (95% CI, 136–198) for surgeries proximal to the thorax along the course of the RLN or the vagus nerve. In post hoc pairwise comparisons of the Kaplan–Meier recovery curves, only the comparison between cardiothoracic surgeries and surgeries proximal to the thorax was statistically significant (adjusted *p* = 0.004 vs. adjusted *α* = 0.0083).

## 4 Discussion

Previously, we found that recovery of both voice and VF motion from iatrogenic UVFP occurred in two phases (i.e., bimodal): an early phase that presumably correlates with recovery from low‐grade neuronal injury and a late phase that correlates with recovery from higher‐grade neuronal injury [1, 2, 3]. The current study parses intubation‐related as distinct from surgically induced UVFP in the iatrogenic category and demonstrates how VF motion recovery time profiles differ between surgically induced, idiopathic, and intubation‐related UVFP. Surgically induced UVFP exhibits a bimodal recovery pattern consistent with the previous model. In particular, the early group appears to regain both voice and VF motion around the same time (overlap of solid and dashed curves in Figure 2A), suggesting functional recovery in an all‐or‐none manner consistent with resolution of a conduction block/low‐grade neuronal injury. For the late group, recovery of VF motion appears to lag voice recovery by about 5 weeks on average. This lag implies recovery of function in a more graded fashion, possibly reflecting regenerating axons successfully traversing the focal injury zone at different times [3]. This concept is more important than the precise amount of time lag, since the 5 weeks is an overestimate depending on when the patients returned for laryngoscopy. It is also worth noting that there is not a clear separation between the early and late groups, with a continuum of mixed recovery existing between the two peaks.

In contrast, both idiopathic and intubation‐related UVFP follow a unimodal recovery model but with very different time courses. Considering idiopathic UVFP first, recovery of VF motion in idiopathic UVFP appears to significantly lag the previously characterized voice recovery time course [2] at first glance, with the peak of the VF motion recovery model at around 20 weeks, and the peak of the voice recovery model at around 6 weeks (Figure 2B). However, the voice recovery curve (fig. 1D in Ref. [2]) was heavily skewed to the right. The median of voice recovery time was 16.4 weeks, compared with the median of VF motion recovery time of 21.9 weeks, again about a 5‐week difference. The median voice recovery time of 16.4 weeks matches perfectly with the median symptom resolution time of 4 months in a large study of idiopathic VFP patients [10]. Our median voice and VF motion recovery times are also congruent with the mean recovery time of 139.4 days reported in a systematic review of idiopathic UVFP by Stonebraker et al. [11], although our voice recovery data [2, 12] did comprise about a third of the data used in that computation.

It is interesting to note that the peak of the VF motion recovery curve for idiopathic UVFP seems to coincide with that for the late group in surgically induced UVFP. We had previously concluded that because the voice recovery profiles of iatrogenic and idiopathic UVFP obviously differed, idiopathic UVFP likely involved a mechanism distinct from a classic focal neuronal injury [2]. However, the apparent overlap in VF motion recovery time courses—at least when compared with surgically induced UVFP beyond Sunderland first‐ and second‐degree injuries—raises the possibility of some mechanistic commonality.

We did note an unusually large subgroup of idiopathic UVFP patients in this study whose first laryngoscopic evidence of motion recovery was noted beyond 34 weeks (Figure 2B). We do not have a good explanation for this observation, as their clinic follow‐up times were not noticeably later than other patients in this study. Sensitivity analysis excluding successive data points in this subgroup did not appreciably alter the model fitting, with the model peak remaining around 20 weeks.

Intubation‐related UVFP exhibits a VF motion recovery model with a single peak around 7 weeks (Figure 2C), overlapping with the peak of the early group in surgically induced UVFP, supporting neuropraxia as the primary mechanism of dysfunction. This mechanism would also explain the faster median time to VF motion recovery and a higher cumulative recovery rate compared with surgical and idiopathic etiologies (Figure 3). What was surprising was that recovery of VF motion could still be prolonged, despite the general expectation that intubation tends to produce compressive neuropraxia [5, 13], which is considered a low‐grade injury. However, longer durations of intubation correspond to longer periods of neuronal ischemia, which can cause Wallerian degeneration and axonal injury [13]. Our model of recovery from a focal neuronal injury strongly suggests that higher grades of injury will require longer recovery times. We suspect that patients who recovered VF motion late had longer duration of intubation; however, the data collected in this study did not include the information necessary to evaluate this correlation. The median time to VF motion recovery was 75 days in our study (Figure 3). This contrasts with the median of 4.3 months reported by Pan and Jiang [5], with the difference likely due to the high proportion of patients intubated for more than 1 day in that series. As an aside, we also note that the proportion of females was much higher for intubation‐related UVFP compared with surgically induced or idiopathic UVFP in this study (Table 1), suggesting greater propensity for endotracheal tube versus laryngeal size mismatch in females than males.

The second objective of this study was to investigate whether the location of injury, that is, distance from the larynx along the RLN, impacted the time to VF motion recovery. Perhaps the most important finding was that for surgically induced UVFP, the time to VF motion recovery clearly did not correlate with the distance between the injury site on the RLN and the larynx. The recovery curves for cardiothoracic surgeries and thyroid/parathyroid surgeries essentially overlap for the first 6 months (Figure 4). This contrasts with the long‐held assumption that recovery time should mirror the presumed RLN regeneration rate of 1 mm/day. This assumption has been called into question by clinical studies that did not show recovery time dependence on site of injury [6, 7], as well as by a controlled study in canines that showed RLN injuries that varied by 5 cm in location recovered the first positive electromyographic signs at essentially the same time [8]. The largest clinical study to date that examined recovery times concluded that a linear association existed between the injury level and the *maximum* recovery time, but their data also showed no difference in the median recovery time based on injury level [14]. Taken together, these findings support a key implication of our previously described two‐phase model for recovery: that the rate‐determining factor in recovery is the severity of injury, not the distance between the injury site and larynx [1]. This derives from modeling the first phase as neuronal regeneration across the injury site as a probabilistic process with an exponential decay distribution, followed by a second phase of unimpeded regeneration back to the larynx as a deterministic process. The tail of the exponential decay drives delayed recovery. We do want to point out that reinnervation via the external branch of the superior laryngeal nerve (SLN) can also explain the equivalence in recovery time as it also does not depend on site of RLN injury, and it is likely that most RLN injuries recover with a mix of recovery pathways that entail reinnervation via both the RLN and the SLN [15].

The major limitation of this study is that the time to VF motion recovery necessarily overestimates the actual time to recovery because it depends on when patients returned for laryngoscopy. This is an intrinsic limitation of retrospective studies on motion recovery, which we attempted to mitigate with an upper bound of 180 days between the first observation of motion recovery and the prior visit. A prospective design with serial exams at predetermined intervals would be required to circumvent this limitation. Second, as pointed out in Section 3, not all patients were followed to full recovery, so the final recovery rates are underestimates, and we did not draw conclusions based on the absolute recovery rates. Despite these limitations, we believe the overall qualitative conclusions remain valid.

## 5 Conclusion

VF motion recovery in UVFP varies by etiology and follows distinct patterns. Surgically induced paralysis demonstrates a bimodal recovery profile, whereas idiopathic and intubation‐related UVFP follow unimodal recovery trajectories with different time scales. Intubation‐related paralysis shows the most rapid and highest overall likelihood of recovery. Across surgically induced etiologies, recovery time did not correlate with the distance between the injury site and the larynx, challenging a long‐held assumption. Instead, these findings support a model in which the severity of neural injury is the principal determinant of time to recovery. These insights may improve prognostic counseling and help guide expectations for recovery following UVFP.

## Funding

The authors have nothing to report.

## Conflicts of Interest

The authors declare no conflicts of interest.

## Supporting information


**Data S1:** Supporting Information.

## Data Availability

The data that support the findings of this study are available from the corresponding author upon reasonable request.
