# Supplementary material for: The Impact of Etiology on Time to Vocal Fold Motion Recovery in Unilateral Vocal Fold Paralysis
Source: Laryngoscope. 2026 Apr 28;136(9):3880–6. doi: 10.1002/lary.70586 (PMC13460803; doi:10.1002/lary.70586)
Supplement: Supplementary file 1 [file LARY-136-3880-s001.docx]

**SUPPLEMENTARY MATERIAL**

**S1. Mathematical Formulation and Model Fitting**

Time to recovery in the early group was modeled as a normal (Gaussian) distribution, while time to recovery in the late group was modeled as an exponentially modified Gaussian distribution. The time to recovery $R\left( t \right)$ is the sum of two probability distributions

$R\left( t \right)=sf\times ER\left( t \right)+LR(t)$	(1)

where $ER\left( t \right)$ is the probability function of the early recovery group,

$ER\left( t \right)=\frac{1}{\sigma_{early}\sqrt{2\pi}}exp\left( \frac{-\left( t-\mu_{early} \right)^{2}}{2{\sigma_{early}}^{2}} \right)$	(2)

and $LR\left( t \right)$ is the probability function for the late recovery group,

$LR\left( t \right)=\frac{1}{\tau}exp\left( \frac{\mu_{late}}{\tau}+\frac{{\sigma_{late}}^{2}}{2\tau^{2}}- \frac{t}{\tau} \right)Ф\left( \frac{t-\mu_{late}-\frac{{\sigma_{late}}^{2}}{\tau}}{\sigma_{late}} \right)$	(3)

$\mu_{early}$ , $\sigma_{early}$ and $\mu_{late}$ , $\sigma_{late}$ correspond to the mean $\mu$and standard deviation $\sigma$ of the Gaussian for the early and late groups, respectively. $\tau$ is the mean duration of the exponential component, and $Ф$ is the cumulative density of the Gaussian function. $sf$ is a scale factor between 0 and 1 and adjusts the relative proportion of the early recovery group vs. the late group. For the current work, $sf$ = 0.6 provided the best fit. For clinical interpretation, $\mu_{early}$ is the mean time to recovery for the early group, $\sigma_{early}$ is the “spread” around this mean; $\mu_{late}$ is the mean time to recovery for the late group, $\sigma_{late}$is the “spread” around this mean; and $\tau$ is how long the “tail” is in the probability distribution.

Time to recovery data was fit to the above two-phase (early/late) model by adjusting the parameters $\mu_{early}$, $\sigma_{early}$, $\mu_{late}$, $\sigma_{late}$, $\tau$, and $sf$. Data fitting was carried out in MATLAB (version R2015a, The MathWorks, Natick, MA) using custom-modified versions of the third-party MATLAB toolbox DISTRIB, which implements a maximum likelihood estimation fitting algorithm for the exponentially modified Gaussian probability distribution function. Details are provided in Mau et al. 2017 (Ref 1).
